# Insights into Mechanisms of Electrochemical Drug Degradation in Their Mixtures in the Split-Flow Reactor

**DOI:** 10.3390/molecules24234356

**Published:** 2019-11-28

**Authors:** Aleksandra Pieczyńska, Stalin Andres Ochoa-Chavez, Patrycja Wilczewska, Aleksandra Bielicka-Giełdoń, Ewa M. Siedlecka

**Affiliations:** 1 Faculty of Chemistry, University of Gdansk, Wita Stwosza 63, 80-308 Gdansk, Poland; Aleksandra.pieczynska@ug.edu.pl (A.P.); p.wilczewska@gmail.com (P.W.); a.bielicka-gieldon@ug.edu.pl (A.B.-G.); 2 Centro de Investigación y Control Ambiental, Departamento de Ingeniería Civil y Ambiental, Escurla Politécnica Nacional, Ladrón de Guevara E11-253, Quito P.O. Box 17-01-2759, Ecuador; stalinandres123@outlook.com

**Keywords:** ifosfamide, cyclophosphamide, 5-fluorouracil, cytostatic drug, BDD anode, electrochemical oxidation, intermediates

## Abstract

The recirculating split-flow batch reactor with a cell divided into anolyte and catholyte compartments for oxidation mixture of cytostatic drugs (CD) was tested. In this study, kinetics and mechanisms of electrochemical oxidization of two mixtures: 5-FU/CP and IF/CP were investigated. The order of the CD degradation rate in single drug solutions and in mixtures was found to be 5-FU < CP < IF. In the 5-FU/CP mixture, k_app_ of 5-FU increased, while k_app_ of CP decreased comparing to the single drug solutions. No effect on the degradation rate was found in the CP/IF mixture. The presence of a second drug in the 5-FU/CP mixture significantly altered mineralization and nitrogen removal efficiency, while these processes were inhibited in IF/CP. The experiments in the different electrolytes showed that •OH and sulphate active species can participate in the drug’s degradation. The k_app_ of the drugs was accelerated by the presence of Cl^−^ ions in the solution. Chlorine active species played the main role in the production of gaseous nitrogen products and increased the mineralisation. Good results were obtained for the degradation and mineralisation processes in mixtures of drugs in municipal wastewater-treated effluent, which is beneficial from the technological and practical point of view.

## 1. Introduction

5-Fuorouracil (5-FU), cyclophosphamide (CP) and ifosfamide (IF) have been the most commonly used cytostatic drugs worldwide since the 1960s. CP has been classified as the most dangerous, carcinogenic compound, meanwhile the 5-FU and IF have been described as compounds with potential mutagenic and teratogenic effects [1,2]. The drugs are polar, poorly biodegradable and under solar light they are photolytic persistent compounds [2,3]. Hospitals and municipal wastewater treatment plants (WWTPs) act as major point sources of cytostatic drugs discharge to the environment, because they are incompletely removed by the mechanical-biological wastewater treatment. The effluents from municipal or hospital wastewater treatment plants generally contain a mixture of different types of pharmaceuticals with their metabolic products.

The efficient removal of cytostatic drugs from wastewater by powerful transformation treatments such as Advanced Oxidation Processes (AOPs) seems necessary to avoid its continuous discharge into waterbodies. However, the reports of investigations of drugs removal by alternative methods in their mixtures have been limited so far. The degradation of a mixture of 16 cytostatic drugs by hydrolysis, photolysis, biodegradation process, and H_2_O_2_/UV system was studied by Franquet-Griell et al. [4]. The physical-chemical and biological processes were selected for the study of the organic compound’s fate during water and wastewater treatment. The results revealed that IF and CP are very persistent compounds in both the natural environment and during wastewater treatment. Another paper reported how mineralization efficiency of chloramphenicol, ciprofloxacin and dipyrone individually and from equimolar mixtures varied in Fenton and photo-Fenton processes. The authors found that degradation efficiency was suppressed by Cl^−^ or F^−^ ions, which were released into the solution upon cleavage of drugs present in the mixture [5]. AOPs including H_2_O_2_/UV or Fenton process are considered promising alternative techniques for the decomposition of poorly biodegradable micropollutants [6,7,8]. However, the application of AOPs to remove pharmaceuticals in wastewater and in single drug solutions led to different levels of effectiveness of individual drugs removal, as was previously proved [9,10,11]. Competition among organic compounds for •OH radical could cause the inhibition or acceleration of the drug oxidation rate in wastewater. Moreover, the inorganic anions e.g., Cl^−^, HCO_3_^−^, can also participate in reactions with •OH radicals, causing a decrease in their concentration available to remove organic matter. The presence of inorganic anions affects the degradation rate of drugs, which depends on their concentration and the type of used AOPs [10,11]. Therefore, the composition of wastewater should be taken into account when assessing the effectiveness of pollution removal by AOPs.

Electrochemical advanced oxidation (EAO) is one of the powerful techniques which are being developed for drugs wastewater remediation [12]. The key factors in the effectiveness of EAO processes are the applied electrode material, the electrolyte composition and the type of cell [13,14]. Among different anode materials, boron-doped diamond (BDD) thin films have attracted great attention. BDD with the weak •OH radicals interaction and the large overpotential for water discharge, enables regulating the yield of generated in situ strong oxidants such as •OH [2]. BDD electrode has been used as an anode for the decomposition of various micropollutants [15] including cytostatic drugs [10]. Another key factor that affects the kinetics and effectiveness of EAO is the type of reactor. A large variety of electrochemical oxidation systems have been tested for the treatment of dyes and drugs wastewater such as: divided or undivided electrode cells, flow cells with parallel electrodes, and flow plants with a three-phase three-dimensional electrode reactor [16,17,18]. It was shown that flow cells with planar electrodes in a parallel plate configuration can increase the oxidation rate of pollutants. Radjenovic and Petrovic applied a BDD electrode in a plate-and-frame electrolytic cell divided by the cation exchange membrane for the anolyte and catholyte compartment to eliminate X-ray contrast media [19].

The aim of this work was to assess whether the EAO in a split-flow reactor with a divided cell of anolyte and catholyte compartments is able to effectively degrade and mineralize cytostatic drugs in mixtures: 5-FU/CP and IF/CP. The impact of electrolyte composition including actual effluents from WWTP on the rate and efficiency of individual drugs oxidation in mixtures: IF/CP and 5-FU/CP was tested. The mechanism of cytostatic drugs degradation was explored through identification of oxidizing species and formed intermediates. To our best knowledge, until now there have been no studies that bring insight into the mechanisms of cytostatic drug removal in a mixture in the split-flow reactor of a divided electrolytic cell.

## 2. Results and Discussion

### 2.1. Comparison of Electrochemical Decomposition in Recirculating Split-Flow Batch Reactor of IF, CF and 5-FU in Single Drug Solution

In the first step of study, cyclic voltammograms for 5-FU, CF and IF (50 mg/L of drug in 42 mM Na_2_SO_4_ and pH of 6.6) at the Si/BDD electrode were recorded. As we suspected, the drugs did not reveal any peaks in the range of potential values from −1.5V to +3.0V, which indicated that electrochemical oxidation of cytostatic drugs in our experimental conditions was possible only in an indirect way by electrogenerated oxidative entities (Figure S1).

Next, in galvanostatic experiments with the recirculating split-flow batch reactor, the time course of the electrochemical degradation of drugs: IF, CF and 5-FU in single drug solution was monitored by HPLC-UV. The initial concentrations of pharmaceuticals used in experiments were 25 mg·L^−1^ and 50 mg·L^−1^. They were employed in order to determine the effect of the presence of additional organic matter in the mixture on the kinetics and mineralization of the individual drug. The concentrations of pharmaceuticals were much higher than that found in the waterbodies, due to minimizing the error in the analysis of inorganic ions released and the intermediates of the drugs oxidation process. The operating parameters: volumetric flow rate (13 L·h^−1^) and current density (150 Am^−2^ (j_app_ > j_lim_)), were previously experimentally selected [20]. The experimental data were well described by pseudo-first-order rate kinetics (R^2^ in range 0.95–0.99). In Table 1, the values of apparent rate constants (k_app_) of 5-FU, CP and IF electrochemical degradation in the single drug solution are listed.

Independently of drug concentration, the degradation rate coefficient (k_app_) value increased in the following order k_app_(5-FU) < k_app_(CP) < k_app_(IF). The k_app_ values were higher for the concentration of 25 mg·L^−1^ (8.9 × 10^−3^–27.7 × 10^−3^ min^−1^) than k_app_ values for the concentration of 50 mg·L^−1^ (3.8–19.1 × 10^−3^ min^−1^) (Table 1). For the lower concentration, the efficiency of the removal of drugs was complete in 300, 150 and 120 min for 5-FU, CP and IF, respectively.

**Figure 1:**
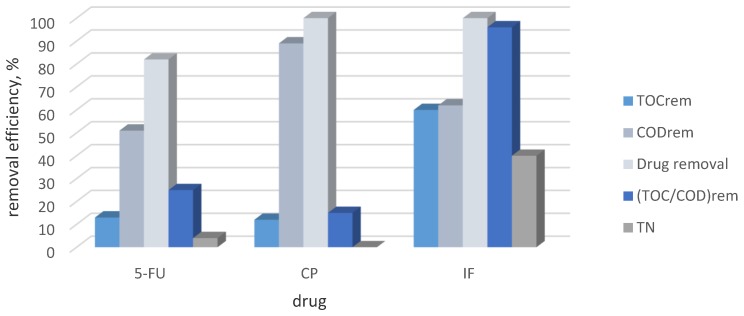
The removal efficiency of TOC, COD, drug concentration, TN and (TOC/COD) ratio after 6 h of electrolysis in single drug solutions in a concentration of 50 mg·L^−1^.

Figure 1 presented the efficiency of the removal of TOC, COD, drug, and TN after 6 h of electrochemical processes. As we can see, TOC conversion reached 13, 14 and 60% respectively for 5-FU, CP and IF indicating that 5-FU and CP were more persistent compounds than IF in the mineralization process. Moreover, the total amount of nitrogen (TN) was still present in the solution in organic or mineral forms for 5-FU and CP, while the TN removal for IF reached 34%. The relatively high value of TN removal for IF degradation indicated that the nitrogen-containing drug was converted to organic and mineral intermediates which existed in the solution, and to gaseous nitrogen species. The release of NOx during mineralization of *N*-containing pollutants by the semi-quantitative method using differential electrochemistry mass spectroscopy was ascertained by Garcia-Segura et al. [21]. The process was found to low extent for 5-FU decomposition and it was not observed for CP degradation. It is worth noting that IF is an isomer of CP, but due to the results it can be suspected that its electrochemical degradation performed in a different way than CP.

The COD/TOC removal ratio characterised the organic matter oxidation progress. The analysis of the parameter shows that, (i) intermediates generated in the electrodegradation of CP were accumulated in solution and (ii) they were higher oxidized compounds than intermediates remaining in the solution and produced in the IF and 5-FU degradation processes. The relatively low TOC conversion (14%) and high COD removal (89%) (TOC/COD_rem_ = 16%) implied that CP was degraded to small molecular carboxylic acids. It is known that direct electron transfer from an organic compound to a BDD electrode is a mechanism of the oxalic acid oxidation [22] and, therefore, the small molecular acids appear to be an inhibitor for their own oxidation and also for other compounds (e.g., HCOOH) that undergo oxidation at high anodic potentials [23,24]. Therefore, the phenomena can inhibit the oxidation of small molecular acids at the BDD anode. The CP degradation to small molecular carboxylic acids was in contrast to IF degradation, where TOC and COD removal were 60 and 62 %, respectively (TOC/COD_rem_ ratio of 96%). In this case, the generated intermediates were mineralized to CO_2_ to a higher extent than in the CP degradation, and the small molecular carboxylic acids were not accumulated in the solution.

**Figure 2:**
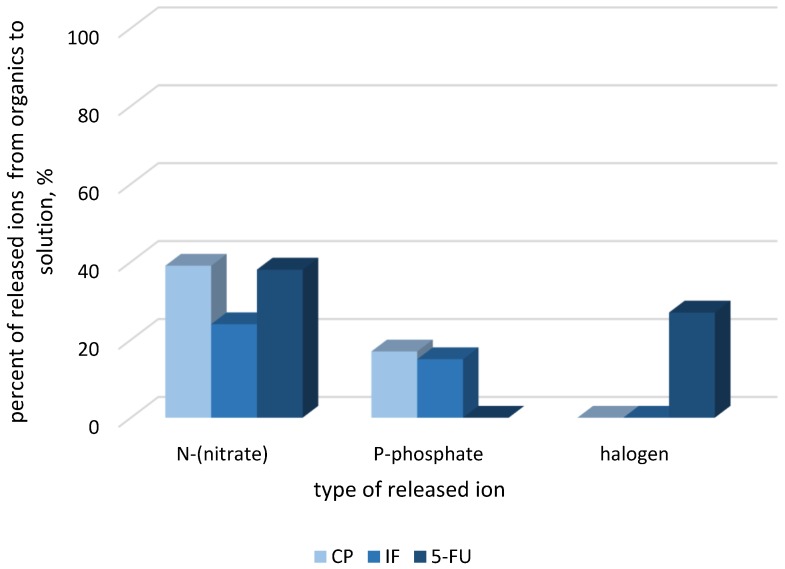
Inorganic ions released in single-drug solution after 6 h of electrochemical oxidation.

The mineralization of IF and CF in the recirculating split-flow batch reactor in the anolytic compartment of the cell was additionally analysed based on the percentage of quantity of mineral products such as Cl^−^, NH_4_^+^, NO_3_^−^ and PO_4_^3−^ released from the organic matter (Figure 2). The amount of inorganic products was expressed as a the percentage of the total expected amount of Cl, N or P released from the initial concentration of each drug. The N-NO_3_^−^ and P-PO_4_^3−^ concentrations were increasing linearly with the time progress, and at the end of the process attained 24 (for IF)-38 (for CP)% and 15 (for IF)-17 (for CP)% of N and P amount initially presented in the drugs solution, respectively. The concentration of N-NH_4_^+^ was very low (below 1 mg·L^−1^) as time passed. A similar trend of Cl^−^ ions releasing was found in our previous study [10]. After 3 h of electrolysis, the amount of Cl^−^ ions reached 50% of total theoretical amount and after that decreased to below detection levels (0.1 mg·L^−1^) for both studied drugs. The results suggested that the organic intermediates existing in the solution after electrolysis still contained in their structures the N and P heteroatoms, while the chloric atoms can be transformed to different forms. Released Cl^−^ ions can be oxidized to Cl_2_ at the anode and, depending on the pH, form HClO/ClO^−^. These chlorine species can undergo electrochemical oxidation to chlorates [25]. Unfortunately, the presence of a high concentration of sulfates in solution (6 g·L^−1^) makes impossible the analysis of a probable trace amount of ClO_3_^−^/ClO_4_^−^ in the effluent and these data cannot be shown. Moreover, the other volatile chlorine species (ClO_2_, Cl_2_O) can be formed. The ClO2 and Cl2O reactions with organic matter do not form significant levels of THMs. They are formed from Cl^−^ in the presence of •OH radicals, mainly in the acid conditions [26]. It is worth noticing that in our experimental conditions in the anolyte compartment, where the oxidation of drugs took place, the pH was about 3.

The 5-FU released to the solution 25% of F^−^ ions and 38% of N-NO_3_^−^, respectively, while the ammonium was in a low concentration (below 1 mg N·L^−1^). The finding suggested that the remaining amount of fluoride and nitrogen was in the organic by-products. The F^−^ ions adsorption onto the BDD electrode surface may also be considered.

### 2.2. Decomposition of Cytostatic Drugs in Their Mixtures

The electrochemical oxidation of mixtures of drugs: CP/5-FU and CP/IF, was investigated. Each compound in the mixture had a 25 mg·L^−1^ concentration. Comparative treatment of drugs in the mixtures (25 mg·L^−1^/25 mg·L^−1^) and in the single compound solutions at two different concentrations (25 and 50 mg·L^−1^) were made in order to clarify the effect of additional organic matter on kinetics and efficiency removal of an individual drug. Moreover, the electrochemical oxidation of mixtures of cytostatic drugs in different electrolytes including effluent from WWTP was examined. The electrochemical experiments were performed in the same operating conditions as described in Section 2.1.

#### 2.2.1. Decomposition of CP and 5-FU Mixture

The decomposition rate of the mixture of drugs was well described by pseudo-first-order rate kinetics. In the CP/5-FU mixture, CP decomposition rate expressed as k_app_ was 11 min^−1^. This value was lower than that found in the single-drug solution, with a concentration of 25 mg·L^−1^ (k_app_ 13.5 min^−1^), and it was similar to the value obtained for CP in the single-drug solution with a concentration of 50 mg·L^−1^. The results indicated that the decomposition of CP was inhibited by concomitantly 5-FU degradation, as an additional organic matter in the solution. On the other hand, the removal rate of 5-FU in the presence of CP was faster than its degradation rate in the single-drug solution regardless of the concentration used (Table 1). The value of k_app_ of 5-FU was likely accelerated by active chlorine species produced from Cl^−^ ions released during CP degradation. In experimental conditions, where the pH of anolyte was strong acid, the electrochemical generation of HClO and Cl_2_•, more selective oxidizing entities than •OH radicals was preferable [27,28].

In order to confirm this hypothesis, the effect of Cl^−^ ions on the drugs mixture decomposition performance was studied. Cl^−^ ions in concentrations of 10 mg·L^−1^ (similar amount of Cl^−^ ions completely released form 50 mg·L^−1^ CP during degradation) and 100 mg·L^−1^ (Table 1) accelerated the rate of both drugs’ oxidation and confirmed the speculation that active chlorine species participated in the degradation of 5-FU even in the presence of a low concentration of Cl^−^ ions released from CP oxidation. During CP decomposition, PO_4_^3−^ ions are also released, therefore, the effects of these ions at concentrations of 5 mgP·L^−1^ (similar to maximal concentration of P released during 50 mg·L^−1^ CP degradation) and 10 mgP·L^−1^ (Table 1) were tested. These ions seems to have an insignificant effect on the electrochemical oxidation of 5-FU and CP.

**Figure 3:**
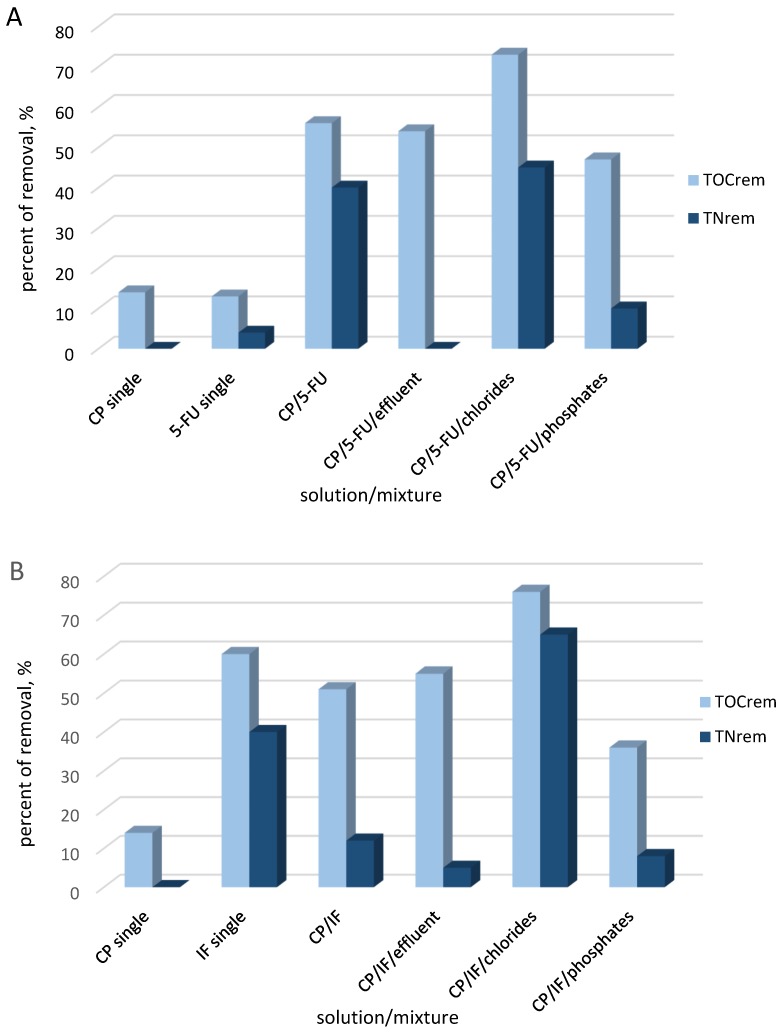
TOC removal and TN conversion efficiency in electrochemical oxidation of single-drug solutions and mixtures of drugs: 5-FU/CP (**A**) and CP/IF (**B**) in different matrices.

In the next step, the mineralization (TOC_rem_) and total nitrogen conversion (TN_rem_ ) to gaseous intermediates were analyzed after 6 h of electrochemical oxidation. TOC and TN results presented in Figure 3A showed that mineralization and nitrogen removal were higher in the mixture compared to the single-drug solutions at a concentration of 50 mg·L^−1^.

The significant increase in efficiency of mineralization and the formation of a higher amount of nitrogen gaseous products in the mixture than in the single-drug solutions were likely the result of two phenomenon: (i) the generation of active chlorine species by Cl^−^ ions being released from CP oxidation and its participation in the decomposition of 5-FU and its intermediates; and (ii) change of the parent compounds’ degradation pathways in the presence of a second organic compound and the generation of less-persistent intermediates for the oxidation process.

The matrix effect was studied in the mixture of 5-FU/CP with the effluent from WWTP. The decomposition rate of drugs was faster in the effluent than in the electrolyte Na_2_SO_4._ The COD was completely removed in the mixture of drugs in the WWT effluent, which is a beneficial from the practical point of view. The TOC conversion of CP/5-FU in the effluent from WWTP was similar to the TOC conversion reached in the mixture of drugs in the Na_2_SO_4_ electrolyte, while the generation of nitrogen gaseous products in the effluent was totally inhibited. The further experiments of CP/5-FU oxidation in the chlorides solution (100 mg·L^−1^) showed the increase of evolution of gaseous nitrogen products and suggested that chlorine active species participated in their generation. In contrast, the phosphates (10 mg·L^−1^) negatively affected this process as well as drugs mineralisation, but to a larger extent inhibited the TN conversion.

We inferred that the faster TOC and TN removal in the presence of Cl^−^ ions in Na_2_SO_4_ was the consequence of the synergy between •OH, SO_4_^−^• radicals and active chlorine species in organic and nitrogen compounds oxidation. The •OH/SO_4_^−^• radicals reacted with the compounds to form the derivatives, which can be more easily attacked by Cl_2_• and HClO. Even the low concentration of Cl^−^ as released form drug degradation is enough to improve the treatment process. The other research group reported that the oxidation potential for the formation of SO_4_^−^• radicals, and their lifetime, show that chlorine active species are more effective in the oxidation of the organic matter in the bulk of the solution [28,29]. The presence of other inorganic ions such as PO_4_^3−^ inhibited this process.

Moreover, depending on the concentration of chlorine formed in direct electrochemical oxidation of Cl^−^ at anode, the ammonium ions transformed to chloramines (Equation (1)) and then to N_2_ and N_2_O (Equations (2) and (3)) in the bulk of the solution were decreasing nitrogen content [30,31]:

NH_4_^+^ + HOCl ↔ NH_2_Cl + H_2_O + H^+^ (1)

4NH_2_Cl + 3Cl_2_ + H_2_O → N_2_O + N_2_ +10 HCl (2)

2NH_4_^+^ + 3HOCl → N_2_ + 3H_2_O + 5H^+^ + 3Cl^−^ (3)

The conversion of amines to nitrates could be a minor pathway (Equation (4))

NH_4_^+^ + 4HOCl → NO_3_^−^ + H_2_O + 6H^+^ + 4Cl^−^ (4)

Comparing the results acquired in the single drug solutions of CP with results obtained in the mixture of CP/5-FU, it was supposed that reaction 4 was possible for electrochemical oxidation of CP in a single-drug solution (Section 2.1). However, the direct oxidation of organic nitrogen by •OH radicals, without previously releasing from CP ammonium ions, could be a more likely pathway of nitrates production [21].

The nitrogen evolution is attained when the chlorine breaking point with the molar ratio Cl_2_:NH_4_^+^ 1:1 was reached. After 6 h of electrolysis of CP solution, most of the organic nitrogen was converted to N-NO_3_^−^ by •OH/SO_4_^−^•, and the value of TN removal was low. The reactions 2 and 3 were favored in the mixture of CP/5-FU, due to the proper ratio of the concentration of NH_4_^+^ and Cl^−^ released from drugs.In the case of the 5-FU single-drug solution, HOCl was absent and •OH/SO_4_^−^• likely participated in the oxidation of both organic nitrogen to N-NO_3_^−^ and organic matter to intermediates and CO_2_.

The results indicated that chlorine active species in the mixture of drugs elevated the 5-FU/CP mineralization and ammonium conversion to nitrogen gaseous products. Thus, the reaction of drugs with active chlorine species can be deemed to be a meaningful transformation mechanism under our experimental conditions.

#### 2.2.2. IF/CP Mixture Decomposition

IF and CF are isomers. However, the quality and quantities of products found during their electrolysis in the bath reactor [10] and their photocatalytic degradation in the presence of Pt–TiO_2_ under solar light was different [11]. Therefore, the examination of the electrochemical decomposition of the CP/IF mixture in the split-flow reactor seems to be interesting.

In the recirculating split-flow batch reactor, the removal rate of IF was twice faster than the CP removal rate. Moreover, the results (Table 1) clearly demonstrated that the degradation rate of the mixture of CP and IF (25 + 25 mg·L^−1^) and the single-drug solutions with a concentration of 25 mg·L^−1^ were similar. It was unexpected that the increase of total organic matter (second drug presence in solution) did not produce any effect on the degradation rate of both drugs. This different behavior in the drug removal process can be related with the presence of different oxidative entities generated during electrochemical process and their participation in cytostatic drugs oxidation.

The PO_4_^3−^ ions slightly inhibited the decomposition of CP, while they did not have any impact on the IF removal. It is possible while the kinetics of oxidants scavenging is slow (e.g., k(SO_4_^−^•+ HPO_4_^−2^) = 1.2 × 10^6^ M^−1^·s^−1^ ). The presence of Cl^−^ ions in the CP/IF mixture mostly increased oxidation of both compounds. The value of k_app_ for CP and IF significantly increased and reached similar values.

The removal efficiency of TOC and TN are presented in Figure 3B. As was found in the mixture of CP/IF, the mineralisation and TN conversion to gaseous products were inhibited comparing to single-drug solutions. After 6 h of electrolysis of the drugs mixture, the nitrogen in the drugs was converted to nitrogen gaseous products to a lower degree than in the IF single-drug solution. In the case of mineralization and TN conversion, the effect of additional organic matter (CP) was observed.

The chlorides significantly elevated CP/IF mixture mineralization and TN conversion to nitrogen gaseous products, while phosphates and WWTP effluents (PO_4_^3−^ 0.2 mg·L^−1^, COD 62 mg·L^−1^, Cl^−^ 92 mg·L^−1^) slightly inhibited the mineralisation process and significantly suppressed the nitrogen removal. This fact implied that the nitrogen removal was the most sensitive process for the radicals scavenging by e.g., phosphates or carbonates. A possible explanation is that the mineralization in the Na_2_SO_4_ electrolyte was related to the formation of •OH radicals, which occur near the electrode surface and in the lower degree sulfate active species which exist in the bulk solution. In the presence of Cl^−^, the indirect oxidation of organic matter and ammonium ions happened by chlorine active species acting in the bulk solution. The scavenging ions such as phosphates especially in the low concentration can inhibit at first the oxidation species in the bulk solution and next the •OH radicals due to the diffusion process. The negative influence on the drugs mineralization of effluents from WWTP was also associated with natural organic matter (COD 62 mg·L^−1^) presence.

Based on the amount of TOC removed, the mineralization current efficiency (MCE) values for each drug and the mixtures were calculated. The order of MCE values was found to be: IF (2.9%) > IF/CP (2.5%) ≈ 5-FU/CP (1.6%) > 5-FU (0.6%) > CF (0.5%). In the case of IF and both mixtures CP/5-FU and IF/CP, the higher the MCE, the less energy was wasted on the side reaction.

### 2.3. Active Species Participated in Cytostatic Drugs Removal

Some authors reported that the Na_2_SO_4_ electrolyte used for oxidation of organic matter at the BDD anode can be the source of •OH and SO_4_^−^• radicals. Compared to •OH, SO_4_^−^• is similarly oxidative (E^0^ (SO_4_•^−^/SO_4_^2^^−^) = 2.5−3.1 V) but more selective toward electron-rich organic contaminants. Therefore, SO_4_•^−^ is more likely to share in electron transfer reactions from the aromatic ring [32], while •OH radicals mainly participate in hydrogen abstraction or addition reactions [33].

In order to check the hypothesis that •OH/SO_4_^−^• took part in electrochemical oxidation of cytostatic drugs in Na_2_SO_4_ electrolyte, the degradation rates of the mixtures of drugs: 5-FU/CP and CP/IF have been compared. Two different electrolytes were applied: NaNO_3_ (42 mM) and Na_2_SO_4_ (42 mM). In the experiments, t-butanol (*t*-but) was used as a scavenger of •OH radicals, because it reacts about 1000 times faster with •OH (6 × 10^8^ M^−1^·s^−1^) compared to SO_4_^−^• (9.1 × 10^5^ M^−1^·s^−1^) [34].

**Figure 4:**
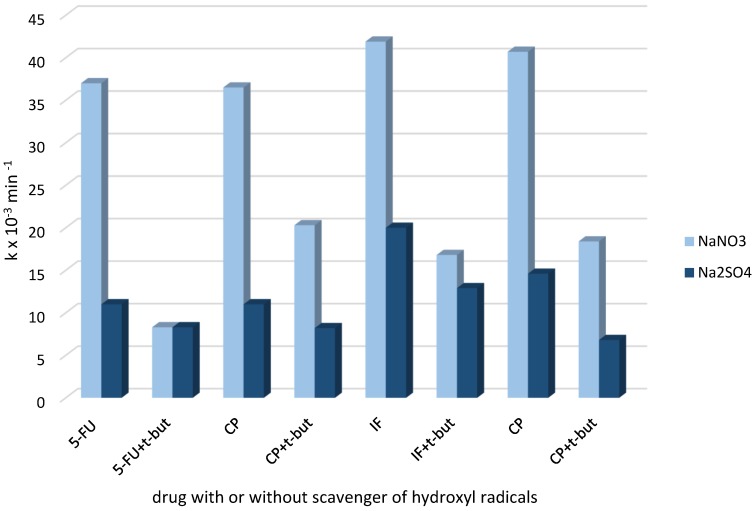
The k_app_ of drugs electrochemical oxidation in their mixtures (5-FU/CP and of IF/CP) in different types of electrolyte with and without t-butanol.

Figure 4 shows the k_app_ of electrochemical oxidation of the mixtures of drugs (5-FU/CP and IF/CP) in different types of electrolytes.

The degradation rate mixtures of drugs in the NaNO_3_ electrolyte were significantly higher than in Na_2_SO_4_. The values of k_app_ were similarly demonstrating that the role of •OH radical in drugs degradation is powerful and the oxidation process is non-selective in this electrolyte. The oxidation process with *t*-but addition to the mixture of 5-FU/CP showed that the degradation rate was more inhibited for 5-FU than for CP. As was expected, the scavenging effect of •OH radicals by t-but in CP/IF mixture resulted in the same suppression of both drugs’ degradation rates. The order of reaction rate of drugs with •OH radicals can be estimated as follows: 5-FU < CP = IF.

In Na_2_SO_4_ electrolyte, the value of k_app_ for IF degradation was higher than values of k_app_ for CP and 5-FU oxidation, (k_app_5-FU = k_app_CP < k_app_IF). Moreover, the degradation of drugs in this electrolyte was less suppressed by OH• scavenger (*t*-but) than in NaNO_3_. The results suggested that in Na_2_SO_4,_ •OH radicals participated in the drug degradation concomitantly with SO_4_^−^• radicals. Long-life SO_4_^−^• occurred in the bulk solution as a product of SO_4_^2−^ ions with the •OH radicals reaction. Consequently, •OH radicals were partially consumed. As it was mentioned previously, SO_4_^−^• radicals react more selectively with organic matter and oxidize drugs less rapidly as •OH radicals. SO_4_^−^• are also less sensitive on the scavenging by *t*-butanol.

In the presence of Cl^−^ ions released in CP and IF degradation, SO_4_^−^• radicals could react with Cl^−^ to produce secondary radicals of Cl• and then Cl_2_^−^• according to the following reactions (Equations (5) and (7)).

SO_4_•^−^ + Cl^−^ → SO_4_^2−^ + Cl•, k = 3.2 × 10^8^ M^−1^·s^−1^ (5)

Cl^−^ + •OH → HOCl, k = 6.1 × 10^9^ M^−1^·s^−1^ (6)

Cl• + Cl• → Cl_2_^−^•, k= 6.5 × 10^9^ M^−1^·s^−1^ (7)

The chlorine active species (HClO, Cl•, Cl_2_•^−^) can also be formed by direct electrolysis of chloride ions [35] released during drugs degradation. The behaviour of cytostatic drugs in mixtures in the Na_2_SO_4_ electrolyte confirms its different reactivity with SO_4_^−^• and/or chlorine active species in contrast to •OH radicals.

**Figure 5:**
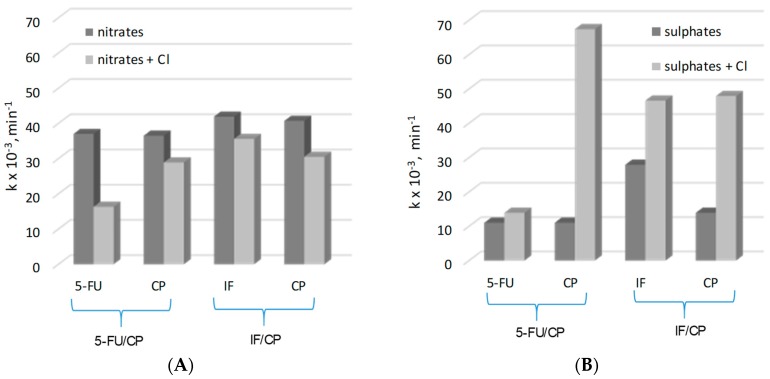
The influence of chlorides on the degradation drugs in 5-FU/CP and CP/IF mixtures in NaNO_3_ (**A**) and Na_2_SO_4_ (**B**) electrolytes.

To improve the speculation that IF, CP and 5-FU react with chlorine active species, the influence of Cl^−^ ions on the drugs degradation in NaNO_3_ and Na_2_SO_4_ electrolytes was compared (Figure 5). In NaNO_3_, chlorides inhibited the degradation of studied drugs, due to the scavenging of OH• radicals and the formation of weaker and more selective oxidants such as Cl• and Cl_2_•. In sulphate, chlorides can promote the SO_4_•^−^ induced oxidation of cytostatic drugs. In Na_2_SO_4_, this can happen because the reaction rate of CP degradation was less inhibited in nitrates and more accelerated in sulphates than the degradation of 5-FU (Figure 5), while 5-FU removal was significantly suppressed by chlorides in the NaNO_3_ electrolyte and slightly accelerated by these ions in Na_2_SO_4_. The conclusion from the results was that the chlorine active species significantly participated in CP degradation, while favourable radicals for 5-FU oxidation were •OH and SO_4_^−^• rather than chlorine active species. Generated BDD electrode chlorine active species play the main role in NH_4_^+^ released from 5-FU oxidation to gaseous products (see Section 2.2.1). The CP/IF mixture’s oxidation in the presence of chlorides in different electrolytes confirmed that IF and CP were oxidized by chlorine active species, but their reactivity was likely different.

In order to study the influence of the type of radicals on the mineralization process, the TOC values in effluents of electrolysis in NaNO_3_ and Na_2_SO_4_ electrolytes were compared. After 3 h of oxidation, TOC removal in NaNO_3_ was 50.8 and 48.0% for CP/IF and 5-FU/CP mixtures, respectively, while TOC removal in these mixtures in Na_2_SO_4_ reached a similar TOC efficiency after 6 h of electrolysis. Due to the high NaNO_3_ concentration (42 mM), TN removal efficiency by •OH radical was not possible to examine in the conditions employed. Based on the results, the •OH/SO_4_^−^• and chlorine active species were effective in cytostatic drugs mineralisation. However, the time needed to obtain the same mineralization efficiency by •OH/SO_4_^−^• and chlorine active species in Na_2_SO_4_ electrolyte as that reached by •OH radicals in NaNO_3_ electrolyte required a twice longer time of electrolysis. In waterbodies, the sulphates and chlorides naturally exist, while nitrates in higher concentration are pollutants and responsible for the eutrophication. Therefore, knowledge about the mechanism of cytostatic drugs degradation in the presence of sulphates, chlorides and phosphates is necessary.

### 2.4. Identification of CP and IF Intermediates in Mixture in Na_2_SO_4_

The intermediates were recorded by LC/MS technique after 2h of CP/IF mixture electrolysis in the Na_2_SO_4_ electrolyte. The intermediates in the CP/IF mixture were found in a higher number but a smaller amount than in the single-drug solutions with a mass number in the range from [M + H]^+^ = 165 to [M + H]^+^ = 311. The intermediates, which were identified both in single-drug solutions and in the mixture were with the molecular weights [M + H]^+^ = 165, 199, 259, 277 and 293. Similar intermediates were reported by other research groups which tested the CP and/or IF degradation by photocatalytic or electrochemical AOPs [10,15,36,37]. Independent of the type of CP and IF solution (single or mixture), the intermediates with mass numbers [M + H]^+^ = 249, 259, 277 and 293 were recognized. The aldophosphamide ([M + H]^+^ = 277) and carboxyphosphamide (([M + H]^+^ = 293) were shown to be the human metabolites of CP and IF. The fact is that carboxyphosphamide had little or no anticancer activity [38]. The structures of possible intermediates identified in the CP/IF mixture are presented in Table 2.

## 3. Materials and Methods

### 3.1. Chemicals

The CP, IF and 5-FU standards were purchased from Sigma-Aldrich (Steinheim, Germany); Acetonitrile (ACN) and the sodium sulphate (supported electrolyte) were obtained from P.P.H. Stanlab (Lublin, Poland). Sodium chloride and monopotassium phosphate were purchased from POCH S.A. (Gliwice, Poland).

### 3.2. Voltammetric Measurements

Cyclic voltammetry analysis was accomplished with the BDD (Adamant Technologies, B/C radio about 500 ppm with a surface of 1 cm^2^) as the working electrode, Ag/AgCl as the reference electrode, and platinum wire as the counter electrode in a solution of 42 mM Na_2_SO_4_ containing 50 mg·L^−1^ of the drug. The experiment was carried out in a 10 mL electrochemical cell, at room temperature. Cyclic voltammograms were performed with a PGSTAT 30 Autolab potentiostat/galvanostat.

### 3.3. Electrochemical Systems

**Figure 6:**
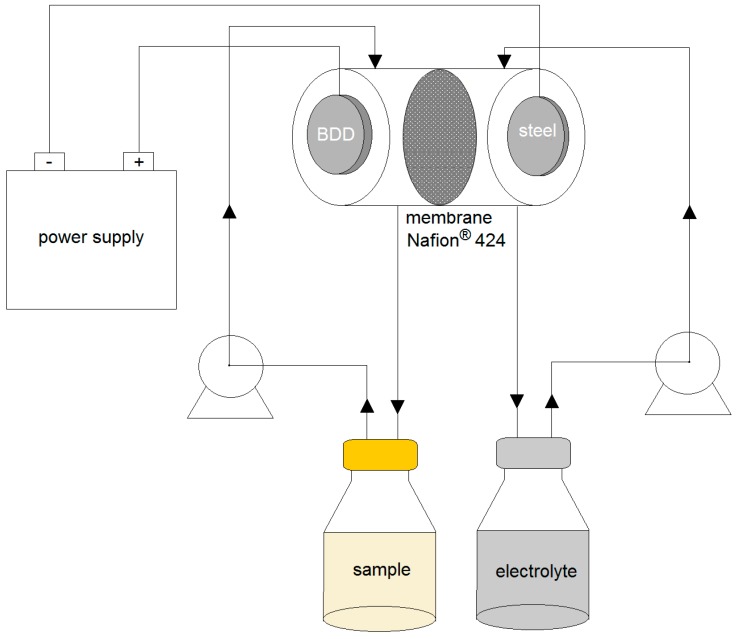
The electrochemical set-up.

Degradation experiments were performed in a recirculating split-flow batch reactor divided into anolyte and catholyte compartments. The cell contains two parallel electrodes, boron-doped diamond (polycrystalline BDD film on monocrystalline *p*-type Si wafer, Adamant Technologies, B/C radio about 500 ppm) used as an anode, and stainless steel (SS) as a cathode. Both were flat (11.7 mm diameter × 2 mm) with an inert-electrode distance of 3.5 cm. Two peristaltic pumps were used to supply the cytostatic drug solution/mixture at constant volumetric flow rate. The total volume of both the anolyte and catholyte was 100 mL. In the divided cell, the anodic and cathodic compartments were separated into two equal spaces by a cation exchange membrane Nafion^®^ 424 to allow the passage of protons. The current was passed through the cell with a potentiostat/galvanostat. Experiments were performed at room temperature 25 °C ± 3 °C. The pH was monitored with the pH-meter. The reactor and glassware used were protected from light. The electrochemical system applied in the study is presented in Figure 6. All experiments were performed in duplicate. The optimal experimental conditions were previously investigated [20]. Electrochemical degradation of the mixtures (IF/CP and CP/5-FU) in the supported electrolyte solution (42 mM Na_2_SO_4_ or 42mM NaNO_3_) was carried out for the initial drug concentration of 25 mg·L^−1^. A comparison between the oxidative processes occurring in the mixtures of these medicaments and single-drug solutions was conducted. The initial concentrations of each drug in a single-drug solution were 25 and 50 mg·L^−1^. Subsequently, the degradation experiment was performed in the same operating conditions with the mixture of the drugs, adding 5 and 10 mg·L^−1^ of PO_4_^3−^ or 10 and 100 mg·L^−1^ of Cl^−^, or with a mixture of drugs added to the actual effluent from the wastewater treatment plan, as seen in Table 3. The following operating conditions were used in the assays: current density of 15 mA·cm^−2^ and flow rate of 13 L·h^−1^. For all kinetics experiments, the drug concentration was monitored by means of the HPLC-UV analysis. The mineralisation process was estimated based on the total organic carbon (TOC) removal and total nitrogen (TN) removal.

The mineralization current efficiency (MCE) according to El-Ghenymy et al. [39] for mixtures of drugs was estimated from Equation (8):

(8) $$\mathrm{MCE}\%=\frac{n\mathrm{FV}\left(\Delta \mathrm{TOC}\right)}{4.32\times 10^{7}\;\mathrm{mIt}}\times 100\%$$

where *n* is the number of electrons consumed per one molecule of drug assuming the total mineralization (based on the reactions Equation (9) for 5-FU and Equation (10) for IF, CF and molar ratio drugs in the mixture), F is the Faraday constant (96,487 C mol^−1^), V (dm^3^) is the solution volume, (ΔTOC) (mg L^−1^) is the difference between TOC before and after electrochemical process, 4.32 × 10^7^ is a factor to homogenize units, m is the number of carbon atoms of drugs, I (A) is the applied current, and t (h) is electrolysis time.

C_4_H_3_N_2_O_2_F + 6H_2_O → 4CO_2_ + 2NH_4_^+^ + F^−^ + 7H^+^ + 8e^−^ (9)

C_7_H_15_N_2_O_2_Cl_2_P + 8H_2_O → 7CO_2_ + 2NH_4_^+^ + 2Cl^−^ + PO_4_^3−^ + 23H^+^ +20e^−^ (10)

### 3.4. Analytical Methods

The concentration of selected drugs was measured by HPLC (Perkin Elmer, Series 200, Shimadzu Europa GmbH, Dulsburg, Germany). The set-up was equipped with a UV detector (SPD-MZOA Shimadzu Dulsburg, Germany) and a C-18 column (Phenomenex 150 × 4.6 nm, 2.6 µm). A detailed description of the analytical methods of CP, IF and 5-FU determination has been reported in our previous works [10].

The values of chemical oxygen demand were measured using standard cuvette tests (HACH) and an Odyssey spectrophotometer. The concentration of chloride was measured by argentometric method, and fluoride (444-49), phosphate (Cadmium reduction), ammonium (Nessler method) and nitrate (21061-69) were measured using cuvette tests (HACH). Additionally, the reaction’s intermediate products were identified with the use of the LC/MS system [37]. The chromatographic parameters were identical with those used during the HPLC-UV analysis. The MS analysis was conducted using positive and negative mode electrospray ionization (ESI) over a mass scan range of 50–350 *m*/*z* (target mass 250 *m*/*z*) under the conditions described in our previous work [37]. TOC and total amount of nitrogen compounds (TN) were measured using TOC/TN analyzer (Shimadzu, TOC-L CSH Dulsburg, Germany).

## 4. Conclusions

In the present work, the electrochemical oxidation of three cytostatic drugs and their mixtures in a recirculating split-flow batch reactor equipped with a BDD anode was investigated. The oxidation of cytostatic drugs was performed in an anodic compartment separated from the cathodic compartment by a cation-exchanged membrane. The values of k_app_ for the single-drug solution and for mixtures of cytostatic drugs in Na_2_SO_4_ as electrolyte were in the following order: k_app_ 5-FU < k_app_ CP < k_app_ IF. The degradation of drugs in their mixtures showed that the PO_4_^3−^ has no significant effect on the drugs’ degradation, while Cl^−^ mainly accelerated this process. Based on the TOC removal and TN conversion to gaseous products, it was found that the degradation pathway of IF was different than that of CP. The organic intermediates found after 6 h of electrolysis were also different for these drugs.

Comparing the results in NaNO_3_ and Na_2_SO_4_ electrolytes, it was suggested that the different oxidising species with different levels of reactiveness participated in cytostatic drugs degradation. In Na_2_SO_4_, 5-FU was mainly oxidized by OH• and SO_4_^−^• radicals, while chlorine active species simultaneously with OH• and SO_4_^−^• entities participated in the CP and IF degradation and nitrogen conversion to gaseous products. Given that Cl^−^ is abundant in natural waters, the involvement of chlorine active species in the electro-oxidation fate of drugs should not be neglected. This was confirmed by the high mineralization and efficiency of drugs removal in the effluent from WWTP, and this is beneficial from the practical point of view. This study is helpful in understanding the fundamental reaction mechanism, as well as the effects of natural water constituents on the kinetics and mechanisms of electrochemical oxidation of cytostatic drugs in their mixtures. Further studies should address the effects of composition of contaminated water (by natural organic matter, carbonates and other anions and cations) on the transformation of cytostatic drugs and the examination of effluents toxicity obtained from AOPs.

## Figures and Tables

**Table 1:** The k_app_ for 5-FU, CP and IF degradation in single drug solutions and in 5-FU/CP and IF/CP mixtures in Na_2_SO_4_ electrolyte.

**Single Drug Solution**	**C = 50 mg L^−1^**		**C = 25 mg L^−1^**			
	**k_app_ × 10^−3^ min^−1^**	**R^2^**	**k_app_ × 10^−3^ min^−1^**	**R^2^**		
5-FU	3.8	0.99	8.9	0.98		
CP	10.4	0.99	13.5	0.99		
IF	19.1	0.99	27.7	0.99		
**Mixture**	**C = 25 mg L^−1^ 1:1**		**C = 25 mg L^−1^ 1:1 + 10 mg L^−1^ Phosphates**		**C = 25 mg L^−1^ 1:1 + 100 mg L^−1^ Chlorides**	
	**k_app_ × 10^−3^ min^−1^**	**R^2^**	**k_app_ × 10^−3^ min^−1^**	**R^2^**	**k_app_ × 10^−3^ min^−1^**	**R^2^**
5-FU/CP						
5-FU	11.0	0.96	10.5	0.97	13.6	0.98
CP	11.0	0.99	10.8	0.96	67.4	0.99
CP/IF						
CP	13.9	0.98	13.0	0.99	47.4	0.95
IF	27.9	0.97	27.7	0.98	46.6	0.99

**Table 2:** The structures of possible intermediates identified in the CP/IF mixture comparing to single-drug solutions.

Drug		Structure of Intermediate	Molecular ion (Fragmentation Ions)
Ifosfamide 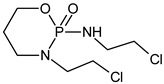	Cyclophosphamide 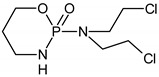		
	✓	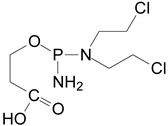	[M + H]^+^ = 293 (275, 227)
✓	✓	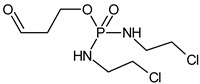	[M + H]^+^ = 277
✓	✓	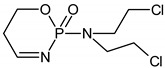	[M + H]^+^ = 259 (140)
✓	✓	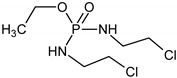	[M + H]^+^ = 249 (164)
✓		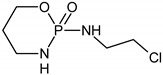	[M + H]^+^ = 199 (171; 79)
✓	✓	?	[M + H]^+^ = 165

**Table 3:** Characteristics of effluents from municipal wastewater treatment plant (MWWTP) with mechanical and biological stages in Gdańsk in Poland.

Parameter	Units	Value
pH		6.8
COD	mgO_2_/L	62
N-NH_4_^+^	mgN/L	0.225
NO_3_^−^	mgN/L	4.463
Cl^−^	mg/L	62
PO_4_^3−^	mgP/L	0.159
SO_4_^2−^	mg/L	89
Conductivity	µS/cm	435

## Supplementary Materials

The following are available online. Figure S1: Cyclic voltammograms of IF, CP and 5-FU (25 mg/L) in 42 mM Na_2_SO_4_ (pH = 6.6), BDD as a working electrode, counter electrode (CE)—Pt, scan rate = 100 mV·s^−1^, T = 20 ± 2 °C.
