# Supplementary material for: Insights into Mechanisms of Electrochemical Drug Degradation in Their Mixtures in the Split-Flow Reactor
Source: Molecules. 2019 Nov 28;24(23):4356. doi: 10.3390/molecules24234356 (PMC6930462; doi:10.3390/molecules24234356)
Supplement: Supplementary file 1 [file molecules-24-04356-s001.pdf]

molecules

Supplementary Materials

Insights into Mechanisms of Electrochemical Drug Degradation in Their Mixtures in the Split-Flow Reactor

Aleksandra Pieczyńska [1], Stalin Andres Ochoa-Chavez [2], Patrycja Wilczewska [1], Aleksandra Bielicka-Giełdoń [1] and Ewa M. Siedlecka [1,*]

[1] Faculty of Chemistry, University of Gdansk, Wita Stwosza 63, 80-308 Gdansk, Poland; Aleksandra.pieczynska@ug.edu.pl (A.P.); p.wilczewska@gmail.com (P.W.); a.bielicka-gieldon@ug.edu.pl (A.B.-G.)

[2] Centro de Investigación y Control Ambiental, Departamento de Ingeniería Civil y Ambiental, Escurla Politécnica Nacional, Ladrón de Guevara E11-253, P.O. Box 17-01-2759 Quito, Ecuador; stalinandres123@outlook.com

* Correspondence: ewa.siedlecka@ug.edu.pl; Tel.: +48-58-523-5228

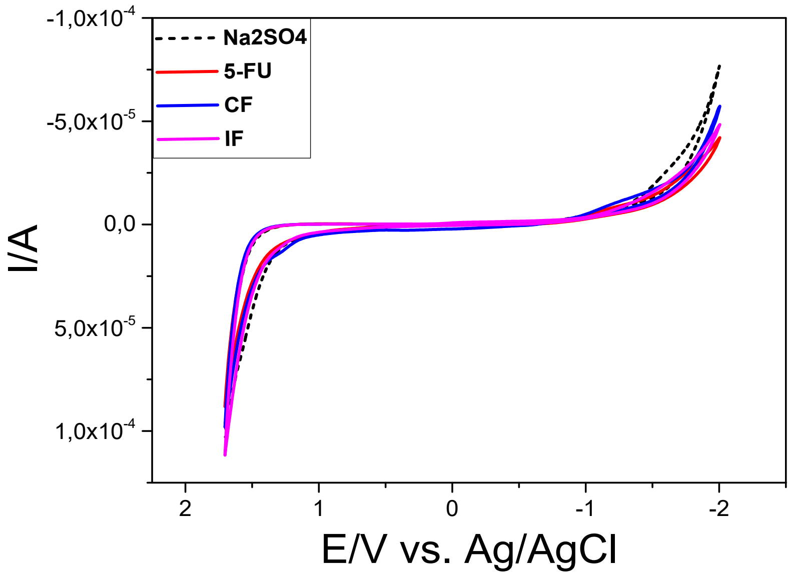

Figure S1. Cyclic voltammograms of IF, CP and 5-FU (25 mg/L) in 42 mM Na_2SO_4 (pH = 6.6), BDD as a working electrode, counter electrode (CE)—Pt, scan rate = 100 mV·s^{-1}, T =20 ± 2 °C.
